# The Hodgkins Fund Prizes

**Published:** 1893-06

**Authors:** 


### The Hodgkins Fund Prizes.

The Smithsonian Institute has issued a circular in reference to these prizes, which are offered for essays on "the nature and properties of atmospheric air in connection with the welfare of man." The prizes are of the value of $10,000, $2,000, and $1,000. Any enquiries concerning the competition should be addressed to S. P. Langley Secretary of the Smithsonian Institute, Washington, D.C., U.S.A.

